# *In Vitro* Anthelmintic Activity and Phytochemical Screening of Crude Extracts of Three Medicinal Plants against *Haemonchus Contortus* in Sheep at Haramaya Municipal Abattoir, Eastern Hararghe

**DOI:** 10.1155/2022/6331740

**Published:** 2022-06-28

**Authors:** Hassen Shamil Mumed, Dereje Regassa Nigussie, Kedir Sali Musa, Abdallahi Abdurahman Demissie

**Affiliations:** Haramaya University, College of Veterinary Medicine, PoB: 138 Dire Dawa, Ethiopia

## Abstract

The current study was carried out to evaluate the anthelmintic efficacy of crude methanolic extracts of leaves of Croton macrostachyus and Nicotiana tabacum and rhizome parts of Zingiber officinale on nematode parasite, Haemonchus contortus. For these objectives, adult worm mortality assay (AWMA) was conducted on adult H. contortus to investigate the in vitro adulticidal effect of crude extracts from March to October 2021 G.C. The percentage yield of crude 99.9% methanolic extracts was 53%, 78%, and 44% for C. macrostachyus, N. tabacum, and Z. officinale, respectively. Phytochemical analysis using standard techniques was also used to detect secondary metabolites contained in the plants. The study revealed the presence of secondary metabolites such as tannins, flavonoids, steroids, and terpenoids in all extracts, which are considered to be the chemical components that are responsible for the wide therapeutic activities of several medicinal plants. In in vitro study, four graded concentrations of the crude extracts (500 mg/ml, 250 mg/ml, 125 mg/ml, and 62.5 mg/ml) were tested at regular time intervals, and parasite viability for 8 hours was recorded in triplicate. Albendazole (1.25 mg/ml) and distilled water were used as the positive and negative controls, respectively. At 4 hr posttreatment, the 62.5 mg/ml, 125 mg/ml, and 250 mg/ml concentrations of C. macrostachyus, N. tabacum, and Z. officinale extracts have caused significantly higher mortality (*P* < 0.05) compared to the albendazole. Methanolic extracts of C. macrostachyus, N. tabacum, and Z. officinale produced mortality of adult H. contortus significantly (*P* < 0.05) to the level of 93%, 83%, and 50% at concentration of 125 mg/ml at 4 hr posttreatment and meanwhile at 6 hr produced 100%, 100%, and 90%, respectively, at the same concentration. On the other hand, albendazole (1.25 mg/ml) killed 60% and 80% of the parasites at 4 hr and 6 hr posttreatment, respectively. Concentrations of all the extracts had showed a comparable and strong nematocidal effect on H. contortus having no significant difference with that of the positive control (*P* > 0.05) at 8 hr posttreatment period. Hence, the current study revealed that the extracts from three plants have potential anthelmintic effect, and we recommend further study on fractionating each component separately and validating the materials using other parasite developmental stages are warranted.

## 1. Introduction

Animal diseases remain a major constraint to livestock productivity across all agro ecological zones and production systems worldwide, especially in small ruminants in tropical and subtropical countries [1]. Among these, helminth infections play a crucial role, mainly in small ruminant production leading to enormous economic losses, particularly loss of production through mortality, weight loss, and reduced milk and meat production [2, 3]. The major classes of endoparasitic helminthes include gastrointestinal parasites which affect ruminants are grouped into cestodes, trematodes, and nematodes. Gastrointestinal nematodes (GIN) of the order Stronglida are the most common causes of clinical helminthosis. These parasites infest the wall or the lumen of abomasum, small intestine, and large intestine. The most common gastrointestinal nematodes of small ruminants belong to the following genera: Haemonchus, Trichostrongylus, Ostertagia, Oesophagostomum, Cooperia, Nematodirus, Marshalagia, Strongyloides and Trichuris [4].

The abomasal nematode, *Haemonchus contortus* (barber's pole worm), which belongs to the family Trichostrongyloidea and genus Haemonchus [5] is a particularly important endoparasite and causes severe anemia and death in severely infected animal. *H. contortus* is a highly pathogenic parasite of small ruminants, and it is the primary constraint to profitable production of sheep and goats worldwide [1, 6]. The disease haemonchosis is especially prevalent in developing countries in association with poor management practices and inadequate control measures [7].

Most of the parasite control programs are based upon a combination of chemotherapeutic control, grazing management, dietary management, biological control, vaccination, and ethnoveterinary treatment [7]. Indeed, chemotherapeutic control is generally achieved by use of synthetic anthelmintics in combination with grazing management [8]. Synthetic anthelmintics have several drawbacks including resistance. *H. contortus* has been documented to be resistant to broad and narrow spectrum families of anthelminthics [9, 10]. Countrywide surveys for anthelmintic resistance have not yet been carried out in Ethiopia [11]. However, studies showed that resistance was detected in different parts of Ethiopia against albendazole, levamisole, tetramisole, and ivermectin [12].

Besides anthelmintic resistance, risk of residue, unavailability, and high cost especially to farmers of low income in developing countries diverted the researchers' attention towards the development of alternate methods for the treatment of haemonchosis [13]. One practical way of developing cheaper and effective anthelmintics is to study indigenous herbal remedies [14]. There have been many reports, mainly from Africa, indicating the effectiveness of plant products against helminth infections in animals [15, 16].

In Ethiopia, medicinal plants and knowledge of their use provide a vital contribution to human and livestock health care needs throughout the country [17]. Available literature surveys clearly show the contribution of medicinal plant as a primary health care options in the country, where 80% of human and 90% of livestock population depend on traditional medicine [18, 19]. The research made so far on Ethiopian medicinal plants has been mostly of producing inventories and checklists, only very few have been touched by modern research which mostly concentrated on medicinal plants against cestodes, and little is done on nematodes [20]. In the current study, three medicinal plants (leaves of *Croton macrostachyus* and *Nicotiana tabacum* and rhizomes of *Zingiber officinale)*, which have anthelmintic properties were selected based on information from traditional healers and available literature surveys with the objectives of detecting the type of secondary metabolites present in crude methanolic extracts of plants using qualitative phytochemical screening methods and investigating *in vitro* anthelmintic activities of crude methanol extracts of three medicinal plants against *H. contortus* using the adult worm mortality (AWM) assay.

## 2. Materials and Methods

### 2.1. Study Area

The study was conducted at Haramaya University College of Veterinary Medicine (HU-CVM), Veterinary Parasitology Laboratory found in Haramaya district, East Hararghe Zone, Oromia National Regional State, Ethiopia. The district is located 508 km East of Addis Ababa (Finfinnee), the capital city of Ethiopia, and 14 km from Harar town to west. Topographically, it is situated at an altitude of 1600 to 2100 above sea level at 09° 24′12^″^ N latitude and 42° 01′ 54^″^ E longitude with the mean annual temperature and relative humidity of 18°C and 68%, respectively, and the area receives an average annual rain fall of approximately 900 mm, with a bimodal distribution pattern. Ecologically, the area has 65% midland and 35% lowland zones. The two predominant soil types are 60% rigo soils and 40% heavy black clay soil. The district has about 76,336 cattle; 65,083 sheep and 84,916 goats; 22,355 donkeys; 356 camels; and 89,800 chickens [21].

### 2.2. Study Design

Experimental study type was conducted from March to October 2021 to investigate *in vitro* anthelmintic activities of crude methanol extracts of three medicinal plants using the adult worm mortality (AWM) assay and also to detect the type of secondary metabolites present in crude methanolic extracts of plants using qualitative phytochemical screening methods.

### 2.3. Plant Collection and Identification

Selection of plants was based on the literature survey on the traditional uses of the plants in Ethiopia and other parts of the world. Those plants with claimed anthelmintic activity but less scientifically evaluated for the purported activity were selected. For *in vitro* evaluation, three medicinal plants were selected based on their priorities in ethnomedicinal uses as claimed by traditional healers. Rhizomes of *Z. officinale* were purchased at Haramaya town, while leaves of *C. macrostachyus* and *N. tabacum* were collected from their natural habitat around “Miidhagaa Tolaa” district and Aweday town, respectively, from March to June 2021. Parts of the plant species were collected and transported to (HU-CVM), Veterinary Parasitology Laboratory. After collection, the plant materials were identified (authenticated), and voucher specimen of each species were deposited at the Herbarium of the Haramaya University, Plant Science Herbarium Department for future reference. List of the plant species used in this study, parts used, and areas of collection are shown in (Table 1).

### 2.4. Extraction Method and Phytochemical Screening

#### 2.4.1. Methanolic Extraction

Sufficient amount of plant materials was collected and brought to the laboratory and washed thoroughly (3 times) with clean tap water. After that, it was air dried completely under shade area at room temperature and then grounded with electric machine grinder separately. The resulting powder was sieved, weighted, and stored in clean stopper bottles and kept in dry place until the extraction process was started. In plant preparation formulation, extraction is the first crucial steps. The powdered plant materials prepared and kept in dried place were subjected to 99.9% methanol as a solvent using maceration technique. Methanol was selected as a solvent for extraction based on the access to the alcohols and general solvent. Methanol was the most preferred solvent for plant extraction possibly owing to its polar nature that ensures the release of several bioactive compounds from plants. It has been scientifically proven that high polarity solvents should be used to extract different bioactive compounds with high accuracy [22]. Fruitful results of active compound in plants mainly depend upon the solvent used for herbal formulation.

The powdered specimen was then subjected to extraction using 99.9% methanol by a cold maceration technique. A total of 300 g of the powdered materials was separately soaked in each extraction solvent (100 g of powder in 500 ml of solvent) followed by electric shaker periodically for 48 hours at 60 RPM of room temperature. The mixture was filtered, and the filtrate was passed through sterile filter paper (Whatman No. 1, Ltd. England). Then, the extract was filtered using rotary evaporator (Laborotal-4001, WB, German) at Haramaya University College of Agriculture, Plant Protection and PhD Research Laboratory, to evaporate methanol at 60°C with 60 RPM and kept overnight in hot air oven at 60°C to obtain the pure crude extracts. The crude extracts were then weighed to know the yield of each plant and recorded in percentage. The resulting extracts were transferred into well-labeled vials and kept in a refrigerator at 4°C until their use for biological and biochemical assays, and the percentage yield was calculated by formula *Yield*(%) = (*W*1∗100)/*W*2, where *W*1 is the weight of the extract residue obtained after solvent removal and *W*2 is the weight of the plant powder.

#### 2.4.2. Phytochemical Screening

Phytochemical screening was carried out to assess the qualitative chemical composition of crude extracts using commonly employed precipitation and coloration reaction to identify the major natural chemical groups and secondary metabolites present in the plants. Combinations of several methods were used to identify the phytochemicals of the medicinal plants. Standard screening tests were conducted using a conventional protocol and reagents on the methanolic extracts of herbs to identify the constituents as described by [23, 24]. The screening was done to detect the presence of bioactive compounds of principle believed to have nematocidal activities: alkaloids, flavonoids, glycosides, phenolic compounds, phlobatannins, saponins, steroids, tannins, and terpenoids. The methods used were chemical tests involving color changes through reaction with different standard reagents. Reagents used and color changes observed are presented in (Table 2).

### 2.5. *In Vitro* Experiments

*In vitro* anthelmintic activity was conducted according to the World Association for the Advancement of Veterinary Parasitology (WAAVP) guidelines [25] with slight modification on parasite collection, preparation, and adult worm mortality assay as described below.

#### 2.5.1. Parasite Collection and Preparation

Adult mature parasites of *Haemonchus contortus* were collected from abomasum of naturally infected sheep slaughtered at Haramaya Municipal Abattoir following the standard procedure as described by [26]. Immediately after death, both ends of the collected abomasums were ligated and transported to the laboratory. Then, it was opened along the greater curvature and its contents emptied into a 4 liter plastic bucket containing 2 liters of water. The parasites were recovered by passing the content through a sieve of 100 *μ*m (micrometer) diameter mesh and picked with wire loop. Adult mature *H*. *contortus* was identified and separated from other parasites based on their morphological characteristics following the keys and description given by [27]. Then, the parasites were collected, washed, and kept in phosphate-buffered saline (PBS) until used for *in vitro* test.

#### 2.5.2. Adult Worm Mortality Assay (AWMA)

Briefly, the experiment was conducted according to [20, 25]. The test was performed in 5 cm diameter glass plastic petri dish. A total of about 420 adult parasites were used in the study. Four concentrations were employed for each plant extract. Ten actively moving worms were placed in each petri dishes filled with 500 mg/ml, 250 mg/ml, 125 mg/ml, and 62.5 mg/ml of the methanolic extracts for each of the three plants materials in distilled water, and distilled water alone with ten actively moving parasites was used as the negative control group in total volume of 4 ml. Albendazole originally dissolved in DMSO (dimethyl sulfoxide) and diluted in distilled water at the concentration of 1.25 mg/ml with ten parasites was used as a positive control. Three replications per each treatment concentration were employed.

The inhibitions of motility of the worms were used as indication of worm mortality or paralysis. Motility of worms was observed, and motile worms were counted at regular time intervals of 0-, 2-, 4-, 6-, and 8-hour posttreatment. Worms not showing any motility were picked out and kept in lukewarm PBS for 10 minutes, and in case of revival in motility, the observed worms were counted as alive; otherwise, they were counted as dead. Death of worms was ascertained by absence of motility for observation period of 5-6 seconds. After 8 hrs, the plant extracts and albendazole were washed away, and the parasites were resuspended in PBS for 30 minutes for possible recovery of the parasite motility. Finally, the number of motile (alive) and immotile (dead) worms were counted under dissecting microscope and recorded for each concentration. The dead worms were easily recognized by their straight flat appearance with no movements at the head and tail regions of the body. The percent mortality of worms was calculated for each extract concentration using the formula: a mortality index was calculated as the total number of dead worms divided by the total number of worms per petri dish.

### 2.6. Data Management and Statistical Analysis

Collected raw data was stored in a Microsoft Excel database system used for data management. SPSS Windows version 20 was used for data analysis. Adult worm mortality (AWM) assay-related result of the study was expressed using descriptive statistics (mean ± SEM, and graph). One-way analysis of variance (ANOVA) followed by Tukey's HSD multiple comparisons was used to compare difference between different *in vitro* groups. All significant levels were set at *P* < 0.05.

## 3. Results

### 3.1. Percentage Extraction Yield and Phytochemical Constituents

The yield of 100 g powder of each plant dissolved with 99.9% methanol was available in (Table 3). Highest percentage yield was detected for *Nicotiana tabacum* with 78% and the smallest one with *Zingiber officinale*, which was 44%.

The phytochemical constituents are shown in (Table 4). Phytochemical screening showed the presence of condensed tannins, flavonoids, steroids, and terpenoids in all extracts.

### 3.2. *In Vitro* Study

#### 3.2.1. Adult Worm Mortality Assay (AWMA)

**Figure 1:**
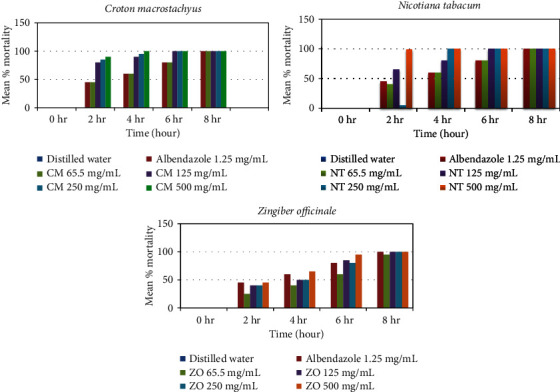
*Mean* ± *SEM* percentage mortality of adult *Haemonchus contortus* after 8 hr posttreatment with four increasing concentrations of methanolic crude extracts of *C. macrostachyus, N. tabacum,* and *Z. officinale* and one concentration of albendazole. Note: CM *= Croton macrostachyus*; NT *= Nicotiana tabacum*; ZO *= Zingiber officinale.*

The present study indicated that all concentrations of methanolic leave part extracts of *C. macrostachyus* and *N. tabacum* as well as the highest concentration of methanolic rhizome extract of Z*. Officinale* produced a relatively comparable anthelmintic activity with the conventional anthelmintic agent, albendazole (Figure 1). The anthelmintic activity of plant extracts increased with time and dose dependent. Accordingly, at 4 hr posttreatment of adult *Haemonchus contortus* to the concentration (62.5 mg/ml, 125 mg/ml, and 250 mg/ml) of extracts, three plants (*C. macrostachyus*, *N. tabacum*, and *Z. officinale*) produced a significant (*P* < 0.05) mortality of adult *H. contortus*. On the other hand, albendazole killed all parasites within 8 hr in concentration of 1.25 mg/ml (Table 5).

**Figure 2:**
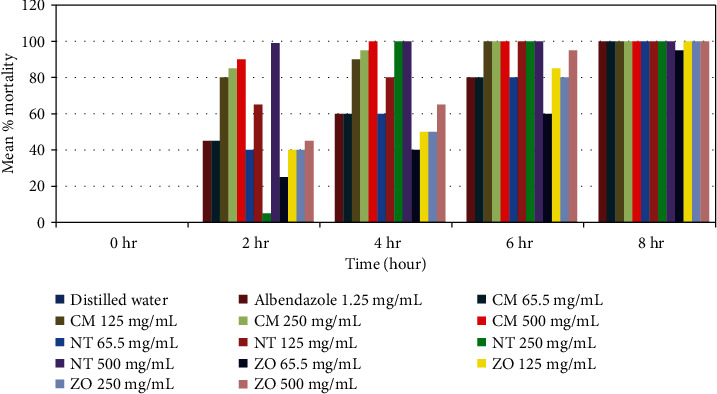
Relative adulticidal efficacy of graded concentration of investigated plant crude extracts using methanolic method and positive control (albendazole, 1.25 mg/ml). Note: CM = *Croton macrostachyus*; NT = *Nicotiana tabacum*; ZO = *Zingiber officinale*.

The overall anthelmintic efficacy of crude extract *in vitro* was statistically significantly different (*P* < 0.05) between them and with positive control. The *relative adulticidal efficacy of graded concentration of extracts* compared with positive control *(albendazole, 1.25* mg*/ml)* is shown in Figure 2.

## 4. Discussion

The problem of anthelmintic resistance, toxicity, and the increasing concern over the presence of drug residues in animal products has led to a renewal of interest in the use of plant-based drugs. Plant materials evaluated in the current study had been identified from various sources to serve as anthelmintic agents by traditional healers of Ethiopia. The *in vitro* tests using free living stages of parasitic nematodes offer a means of evaluating the anthelmintic activity of new plant compounds [28]. In vitro techniques are preferred to in vivo methods due to their low cost, simplicity, and rapid turnover [29]. Moreover, for in vitro studies, *Haemonchus contortus* is proved to be a good test worm because of its longer survival in PBS. This abomasal helminth has recently been used for in vitro studies by other workers [30, 31].

This study revealed that there was a difference yield percentage of extracts among the plants. The leaf of *N. tabacum* presented the highest yield (78%) among extracts followed by *C. macrostachyus* (53%), while the lowest (44%) was observed for the rhizome of *Z. officinale* (Table 3). This finding was in contrary with [32] who reported 22% and 29.3% methanolic leaf extract of *N. tabacum* and *C. macrostachyus*, respectively. Some differences on the percentage yield of these extract materials among the plants might be due to the difference on the nature of plant species, chemical composition differences of the extracts, different environmental conditions which create differences in phytochemical constitution, and harvest time. Furthermore, the solvents and test protocols used during extraction promote difference in concentrations and classes of secondary bioactives present in extracts [33].

In the phytochemical screening test, *C. macrostachyus* (leaf) was found positive for flavonoids, glycosides, phenolic compounds, phlobatannins, saponins, tannins, steroids and terpenoids while negative for alkaloids (Table 4). This finding is inconsistent with [32] who reported the presence of alkaloids, saponins, tannins, phenolic compounds, steroids, and glycosides and meanwhile absence of phlobatannins, flavonoids, and triterpenes. The crude extract of *N. tabacum* (leaf) was positive for alkaloids, flavonoids, phenolic compounds, saponins, tannins, steroids, and terpenoids while negative for glycosides and phlobatannins which is in line with a similar study by [34] who reported the presence of saponins. Other study showed that extract of *N. tabacum* leaves was found positive for alkaloids, saponins, and tannins [35, 36]. This finding is inconsistent with Jelalu et al. [32] who reported the absence of saponins and glycosides. Differences in climatic conditions, tests used, cultivation, and collection of plant materials for extract production may cause differences in results [37]. Growth conditions of the plants also determine the types and quantities of secondary metabolites derived from natural sources that are subject to variability [38].

The current study revealed that the mean mortality of adult *Haemonchus contortus* was increased significantly with increased dosage (concentration) and exposure time after *in vitro* treatment for the tested botanicals. This result is in line with the findings of [39, 40] in which mortality effect of botanicals was indicated to be dosage (concentration) and exposure time dependent.

The *in vitro* adult *Haemonchus contortus* mortality activity of methanolic leaf extracts of *C. macrostachyus* is increased with increased concentrations and time exposure (Table 5). After 4 hr postexposure, *C. macrostachyus* showed high mortality (80%) at concentrations of 62.5 mg/ml which was comparable with albendazole, 1.25 mg/ml (80%). All concentrations of *C. macrostachyus* showed adult *H. contortus* mortality effects at different concentrations and time exposure compared with the negative control. The present result is comparable with those obtained by other scholar. [20] reported that the hydroalcoholic extracts of *C. macrostachyus* (seeds) had mortality effect of 90% at 8 mg/ml after 24 hr posttreatment, while the aqueous extract had 36.67% mortality against *H. contortus*. Differences among these studies might be due to the difference in solvent used for extraction as studies have shown that organic solvent extracts show greater biological activity than the aqueous extract [41].

Similar to *C. macrostachyus*, *N. tabacum* showed good *in vitro* adult *Haemonchus contortus* killing effect. As concentration and exposure time increased, mortality of adult *H. contortus* was also increased. At the highest concentration (500 mg/ml), the killing effect of the plant was higher than the positive control. All concentrations of *N. tabacum* including the least concentrations had effects on Haemonchus when compared with the negative control (Table 5). Besides, especially for concentrations of 500 mg/mL, 250 mg/mL, and 125 mg/mL, more than 50% of mortality was observed as early as 4 hr postexposure to the extracts. Overall, a positive correlation was noted between graded concentrations of the extracts, the exposure test-time interval (Figure 1), and *in vitro* adulticidal effect (Figure 2). The rhizome extracts of *Z. officinale* showed moderate antinematocidal activity against *Haemonchus contortus* at higher concentration (500 mg/mL) and have little activity at least concentration (62.5 mg/mL) after 4 hr postexposure. Comparative *in vitro* nematocidal activity of crude extracts of the plants revealed higher mortality of adult Haemonchus exposed to *C. macrostachyus* (100%) and *N. tabacum* (100%) compared with *Z. officinale* (93.33%) after 8 hrs of exposure at 62.5 mg/mL concentration. The di?erence in mortality percentage of these plants might be due to variability on the amount of secondary metabolites among the plant extracts. The phytochemical analysis in this study showed that *C. macrostachyus* has more secondary metabolites than others. In this study, the relatively lower nematocidal activity of *Z. officinale* to *C. macrostachyus* and *N. tabacum* might be due to lower quantity of secondary metabolite. The high efficacy of *N. tabacum* against *H. contortus* could be attributed to the presence of nicotine, a ganglion stimulant [42]. Nematode muscles are known to contain excitatory neuromuscular junctions containing ganglion type nicotinic receptors with acetylcholine as their neurotransmitter [43]. Any ganglion stimulant would tend to activate these neuromuscular junctions causing a spastic paralysis in the worms leading to their death and expulsion from the host [44].

## 5. Conclusion and Recommendations

The findings from the current study revealed that the phytochemical screening of the three medicinal plants showed the presence of different secondary metabolites which is conserved as a potential source of anthelmintics. Besides, extracts from plants like *Croton macrostachyus*, *Nicotiana tabacum*, and *Zingiber officinale* have shown promising *in vitro* anthelmintic activity against adult *Haemonchus contortus*, which supports the traditional use of these plants as anthelmintics. Efficacy of the extracts increases with increasing concentration and exposure time.

## Figures and Tables

**Table 1:** Species, herbariums voucher no., areas of collection, and parts of medicinal plants used in the current study.

Species (family)/scientific name	Local name	Voucher no.	Area of collection	Parts used
*Croton macrostachyus* (Euphorbiaceae)	Bakkanniisa	002863	Miidhagaa	Leave
*Nicotiana tabacum* (Solanaceae)	Tamboo	006912	Aweday	Leave
*Zingiber officinale* (Zingerberaceae)	Jinjibil		Haramaya	Rhizome

**Table 2:** Phytochemical screening methods (Debella, 2002; Harborne, 2007).

Secondary metabolites	Phytochemical test	Chemicals/method	Indicator
Alkaloids	Mayer's test	Mayer's reagent	Creamy precipitate
Flavonoids	Shinoda's test	NaOH+HCl (dilut.)	Intense yellow/colorless
Glycosides	Ferric chloride test	20%KOH + %5FeCl_3_	Black precipitate
Phenolic compounds	Ferric chloride test	1%FeCl_3_+ 1 ml of K_3_Fe(CN)_6_	Bluish green color
Phlobatannins	HCl test	2 ml of 1%HCl	Deposition of a red precipitate
Saponins	Frothing test	Heat	Formation of persistent honeycomb froth
Steroids	Salkowski's test	CHCl_3_+H_2_SO_4_ (conc.)	Reddish brown color
Terpenoids			Formation of yellow color
Tannins	Ferric chloride test	0.1%FeCl_3_ (dilut.)	Brownish green or blue black color

Note: CHCl_3_ = methyl chloroform; Conc. = concentrated; FeCl_3_ = ferric chloride; H_2_SO_4_ = sulfuric acid; HCl = hydrochloric acid; K_3_Fe(CN)_6_ = potassium ferrocyanide; KOH = potassium hydroxide; NaOH = sodium hydroxide.

**Table 3:** Percentage yield of the study plants using methanolic extraction method.

Plant name	Sample taken in gram	Yield in gram	% yield in (*W*/*W*)
*Croton macrostachyus*	100	53	53
*Nicotiana tabacum*	100	78	78
*Zingiber officinale*	100	44	44

**Table 4:** Qualitative phytochemical constituents found in investigated plant extracts using methanolic method.

Phytochemicals	*C. macrostachyus*	*N. tabacum*	*Z. officinale*
Alkaloids	—	++	+
Flavonoids	++	+	+
Glycosides	+	—	—
Phenolic compounds	++	++	—
Phlobatannins	+	—	+++
Saponins	+	++	—
Tannins	++	++	+
Steroids	++	++	++
Terpenoids	+	++	++

Note: **+++** = the highest constituent; ++ = moderate constituent; + = the latest constituent; **—** = not found.

**Table 5:** Mean adult worm mortality (±SEM) of different concentrations of the crude extracts and positive control (albendazole, 1.25 mg/ml) on adult mortality assay of *H. contortus* for 8-hour posttreatments.

Treatment	Concentration (mg/ml)	Number of parasites dead posttreatment (hours)				
		0 hr	2 hr	4 hr	6 hr	8 hr
*Croton macrostachyus* (leaves part)	500	0.00 ± 0.00	9.33 ± .667^ade^	10.00 ± .000	10.00 ± .000^ab^	10.00 ± .000^a^
	250	0.00 ± 0.00	8.67 ± .667^ade^	9.67 ± .333^bade^	10.00 ± .000^ae^	10.00 ± .000^a^
	125	0.00 ± 0.00	7.67 ± .333^ade^	9.33 ± .333^bade^	10.00 ± .000^ab^	10.00 ± .000^a^
	62.5	0.00 ± 0.00	4.67 ± .333^ade^	6.33 ± .333^bade^	8.00 ± .000	10.00 ± .000^a^
*Nicotiana tabacum* (leaves part)	500	0.00 ± 0.00	9.67 ± .333^ace^	10.00 ± .000	10.00 ± .000^ab^	10.00 ± .000^a^
	250	0.00 ± 0.00	8.67 ± .333^ace^	10.00 ± .000^abce^	10.00 ± .000^ace^	10.00 ± .000^a^
	125	0.00 ± 0.00	6.67 ± .333^ace^	8.33 ± .333^abce^	10.00 ± .000^ab^	10.00 ± .000^a^
	62.5	0.00 ± 0.00	3.67 ± .333^a^	6.33 ± .333^abce^	8.33 ± .333^ace^	10.00 ± .000^a^
*Zingiber officinale* (rhizome part)	500	0.00 ± 0.00	4.67 ± .333^cad^	7.00 ± .000	9.33 ± .333^ab^	10.00 ± .000^a^
	250	0.00 ± 0.00	3.67 ± .333^cad^	5.00 ± .000^cabd^	8.33 ± .333^cabd^	10.00 ± .000^a^
	125	0.00 ± 0.00	3.67 ± .333^cad^	5.00 ± .000^cabd^	9.00 ± .000^ab^	10.00 ± .000^a^
	62.5	0.00 ± 0.00	2.67 ± .333^ca^	4.00 ± .000^cabd^	6.33 ± .667^cabd^	9.33 ± .333^a^
Albendazole	1.25	0.00 ± 0.00	4.67 ± .333^a^	6.00 ± .000^acde^	8.00 ± .000^acde^	10.00 ± .000^a^
Distilled water	00.00	0.00 ± 0.00	0.00 ± 0.00^cbde^	0.00 ± 0.00^cbde^	0.00 ± 0.00^cbde^	0.00 ± 0.00^cbde^

Note: values are mean ± SEM. All superscripts indicate significance at *P* < 0.05. ^a^Compared to untreated (DW), ^b^compared to albendazole, and ^c^compared to each concentration of methanolic extract of *C. macrostachyus* leave part; ^d^compared to each concentration of methanolic extract of *N. tabacum* leave; and ^e^compared to each concentration of methanolic extract of *Z. officinale* rhizome part.

## Data Availability

Upon reasonable request, the supporting dataset is available from the corresponding author. Moreover, the dried specimen of the tested plants and their voucher numbers are deposited in the herbarium of Haramaya University.
